# Embolization with Histoacryl Glue of an Anastomotic Pseudoaneurysm following Surgical Repair of Abdominal Aortic Aneurysm

**DOI:** 10.1155/2013/761384

**Published:** 2013-02-12

**Authors:** Ayesha Walid, Tanveer Ul Haq, Raza Sayani, Zia Ur Rehman

**Affiliations:** ^1^Radiology Department, Aga Khan University Hospital, P.O. Box 3500, Karachi 74800, Pakistan; ^2^Vascular Surgery Department, Aga Khan University Hospital, Karachi, Pakistan

## Abstract

We report a 62-year-old female who had surgical repair of abdominal aortic aneurysm with a bifurcated graft 2 years ago. She presented with a distal anastomotic pseudoaneurysm which was successfully embolized with histoacryl glue. Only one such similar case has been reported in the literature so far (Yamagami et al. (2006)).

## 1. Introduction

Pseudoaneurysm formation is one of the rare, late occurring complication of aortic reconstructive surgery [1]. Once diagnosed, this has to be treated as a potentially lethal complication. Surgical treatment is associated with a high perioperative morbidity and mortality. Minimally invasive endovascular treatment is therefore a safe alternative to surgery [2].

Endovascular stent graft placement and coil embolization are well-established techniques, used alone or in combination with histoacryl glue. We present a case in which histoacryl glue was used alone and successfully embolized distal anastomotic pseudoaneurysm.

## 2. Case Report

**Figure 1:**
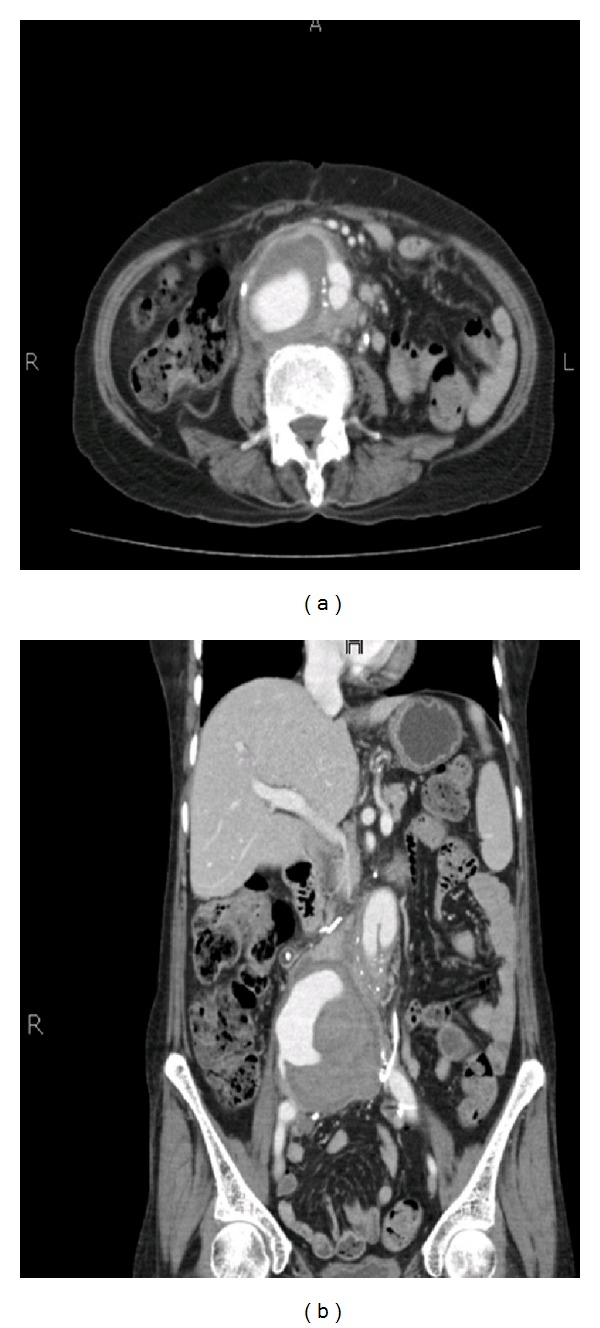
A 62-year-old woman with prior history of surgical repair of abdominal aortic aneurysm with a bifurcated graft presented with distal anastomotic pseudoaneurysm. Enhanced CT obtained 2 years after surgery reveals a pseudoaneurysm of 7.8 cm in diameter with pooling of IV contrast within it, close to the distal anastomosis.

The patient was a 62-year-old female, a known hypertensive and asthmatic. She was diagnosed with an abdominal aortic aneurysm 2 years ago and had elective surgical repair with a bifurcated graft. The proximal end of the graft was anastomosed with infrarenal aorta, and the graft limbs were anastomosed with the common iliac arteries bilaterally. She was hospitalized twice during this period with urinary tract infection. An unenhanced CT KUB examination was performed 2 years following the surgical repair of the abdominal aorta. This showed soft tissue thickening within the retroperitoneum along the right side of the distal abdominal aorta, raising the possibilities of hematoma/retroperitoneal fibrosis. The patient also had bilateral hydronephrosis and hydroureter likely to be secondary to ureteric strictures as postsurgical sequelae. Subsequently the patient developed episodic left flank and leg pain with nausea and vomiting. She was investigated 2 months later with a contrast-enhanced CT abdomen examination that revealed a large pseudoaneurysm measuring 10 cm in diameter, near the distal anastomosis. The pooling of IV contrast was also seen within the pseudo aneurysm suggesting active leak (Figure 1).

**Figure 2:**
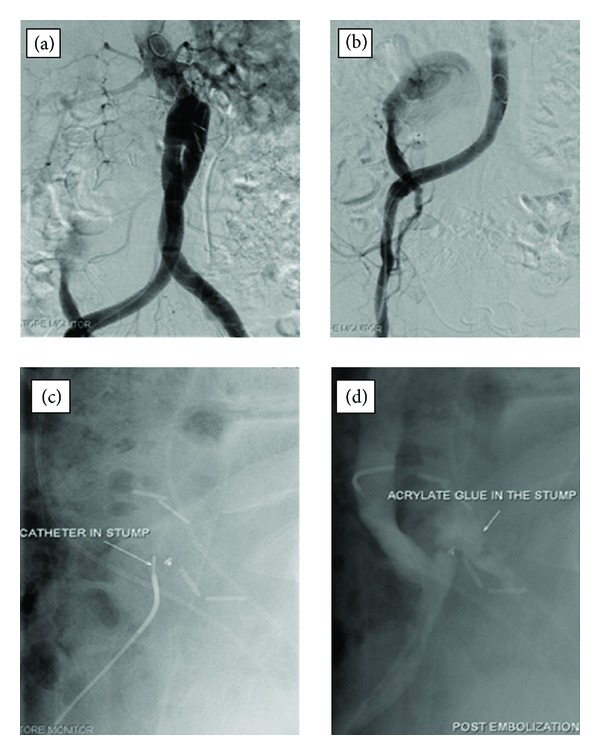
(a) Aortogram shows pooling of the leaked contrast agent from the distal anastomosis. (b) Image obtained with tip of the microcatheter positioned at the ostium of the pseudoaneurysm (arrow). (c, d) Acrylate glue seen within the pseudoaneurysm on postembolization image (arrow).

Subsequent digital subtraction angiogram was done which showed active contrast leak from the distal aortic surgical anastomosis on the right side (right iliac artery). Contrast was seen filling up a pseudo aneurysm measuring approximately 10 cm in size. Initially the option of using endovascular stent graft was considered, and then deferred due to the associated later risk of endoleak. It was decided to embolize the pseudoaneurysm with histoacryl glue. Microcatheter was subsequently advanced, its tip positioned at the ostium of the pseudoaneurysm, and histoacryl glue mixed with lipiodol was injected as the embolic agent. Extreme care was taken not to overflow the embolic agent into the iliac artery. Postembolization angiogram revealed complete exclusion of the pseudoaneurysm. No complications occurred during the procedure (Figure 2). 

**Figure 3:**
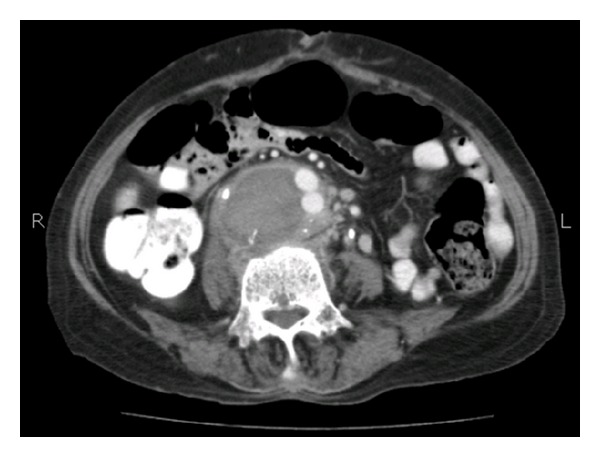
Enhanced CT obtained 15 days after embolization shows that the pseudoaneurysm is thrombosed.

Followup CT examination 15 days later showed thrombosed pseudoaneurysm with reduction in size and no active contrast extravasation (Figure 3).

## 3. Discussion

Standard open abdominal aortic reconstructive surgeries are associated with complications [2]. Pseudoaneurysm formation at the surgical anastomotic site is a relatively rare but a serious, late occurring complication. Plate et al. have reported a frequency of 1.3% among 1087 patients after surgical repair of abdominal aortic aneurysm [1]. Pseudoaneurysms are the result of disruption of the graft-to-vessel anastomosis with actual defect within the arterial wall [3]. These develop more commonly at the distal anastomotic site, usually in the groin, and therefore being symptomatic, they are easily diagnosed. Pseudoaneurysms at the proximal anastomosis are less frequent, and they tend to be asymptomatic until they rupture [2].

Once a pseudoaneurysm ruptures, the mortality rate is very high (96.2%); therefore, these need to be treated as a potentially lethal complication [1].

Surgical treatment is not only challenging due to adhesions from a prior surgery but they are also associated with high perioperative morbidity and mortality. Minimally invasive endovascular treatment is therefore a safe and a feasible alternative to surgery [2]. One of the options in endovascular treatment is the use of a stent graft placement for the exclusion of the pseudoaneurysm from the systemic circulation. This has a high reported technical success rate of 97–100% [2] but is also associated with potential complication of endoleak which can result in continued aneurysmal growth and potential rupture [4].

Platinum coils can be used as an embolic agent for embolization of anastomotic pseudoaneurysm. This is however laborious, time consuming and often requires placement of multiple coils making it expensive [5]. These may even get dislodged. Histoacryl glue is a tissue adhesive which is used for AVMs embolization in brain and so forth. It is unique in that as a monomer, it is a liquid and can be delivered easily through the narrow lumen catheters. When it comes in contact with ions in blood, it rapidly polymerizes, solidifies, and forms a vascular occlusion plug [6]. Histoacryl is mixed in a ratio of 0.5 cc of glue to 1.0–1.5 cc of lipiodol oil, which not only makes the embolized vessel visible but it also helps to regulate the adhesive time [7].

This technique was successfully employed in our case taking extreme care that histoacryl glue does not overflow into the iliac artery and thus avoiding the potential complication of organ infarction.
